# Parental Factors Associated with COVID-19 Vaccine Uptake for Children over 5 Years of Age in Texas

**DOI:** 10.3390/vaccines12050526

**Published:** 2024-05-11

**Authors:** Paula M. Cuccaro, Jihye Choi, Yordanos M. Tiruneh, Journey Martinez, Jing Xie, Michelle Crum, Mark Owens, Jose-Miguel Yamal

**Affiliations:** 1 Center for Health Promotion and Prevention Research, School of Public Health, The University of Texas Health Science Center at Houston, Houston, TX 77030, USA; jihye.choi@uth.tmc.edu; 2 Department of Health Promotion and Behavioral Sciences, School of Public Health, The University of Texas Health Science Center at Houston, Houston, TX 77030, USA; 3 Department of Preventive Medicine and Population Health, School of Medicine, The University of Texas at Tyler Health Science Center, Tyler, TX 75708, USA; yordanos.tiruneh@uttyler.edu (Y.M.T.); michelle.crum@uttyler.edu (M.C.); 4 Division of Infectious Diseases, Department of Internal Medicine, The University of Texas Southwestern Medical Center, Dallas, TX 75390, USA; 5 Coordinating Center for Clinical Trials, School of Public Health, The University of Texas Health Science Center at Houston, Houston, TX 77030, USA; journey.martinez@uth.tmc.edu (J.M.); jing.xie@uth.tmc.edu (J.X.); jose-miguel.yamal@uth.tmc.edu (J.-M.Y.); 6 Department of Biostatistics and Data Science, School of Public Health, The University of Texas Health Science Center at Houston, Houston, TX 77030, USA; 7 Department of Political Science, The Citadel, Charleston, SC 29409, USA; mowens6@citadel.edu

**Keywords:** COVID-19, vaccination, vaccine hesitancy, intention, parent, child

## Abstract

The COVID-19 vaccine is safe and effective for children, yet parental hesitancy towards vaccinating children against the virus persists. We conducted a telephone-administered weighted survey in Texas to examine parents’ sociodemographic factors and medical conditions associated with COVID-19 vaccination intention for parents with unvaccinated children ages 5–17 years. We collected responses from 19,502 participants, of which 4879 were parents of children ages 5–17 years. We conducted multiple logistic regression with Lasso-selected variables to identify factors associated with children’s vaccination status and parents’ intention to vaccinate their children. From the unweighted sample, less than half of the parents (46.8%) had at least one unvaccinated child. These parents were more likely to be White, English-speaking, not concerned about illness, privately insured, and unvaccinated for COVID-19 themselves (*p* < 0.001). In the adjusted regression model, parents who were unvaccinated (vs. having COVID-19 booster, aOR = 28.6) and financially insecure (aOR = 1.46) had higher odds of having unvaccinated children. Parents who were Asian (aOR = 0.50), Black (aOR = 0.69), Spanish-speaking (aOR = 0.57), concerned about illness (aOR = 0.63), had heart disease (aOR = 0.41), and diabetes (aOR = 0.61) had lower odds of having unvaccinated children. Parents who were Asian, Black, Hispanic, Spanish-speaking, concerned about illness for others, and vaccine-boosted were more likely to have vaccination intention for their children (*p* < 0.001). Children’s vaccination is essential to reduce COVID-19 transmission. It is important to raise awareness about the value of pediatric COVID-19 vaccination while considering parents’ sociodemographic and medical circumstances.

## 1. Introduction

The coronavirus disease 2019 (COVID-19) pandemic has posed a significant threat not only to adults but also the pediatric population. As of May 2023, more than 15 million children in the United States (US) have tested positive for COVID-19 [1], having both immediate and long-lasting impacts on their physical, mental, and social well-being [2,3]. COVID-19 has become a leading cause of death for children in the nation [4]. The immaturity of the respiratory tract and immune system in children may considerably increase their susceptibility to COVID-19, especially with the emergence of new variants [5]. Although children exhibit lower rates of COVID-19-related morbidity and mortality compared to adults, they can be facilitators of viral transmission, posing risks to vulnerable populations, such as seniors [6,7]. Given the adverse consequences of pediatric COVID-19 infection, timely uptake of the COVID-19 vaccine among children is essential. A safe and effective pediatric COVID-19 vaccine can protect children from viral infection, and especially mitigate hospitalization rates among children with underlying comorbidities [8,9]. However, parental acceptance of COVID-19 vaccination for their children remains a public health concern [10].

The US Food and Drug Administration (FDA) approved the Pfizer-BioNTech COVID-19 vaccine for emergency use in children aged 12–15 years and 5–11 years in May and October 2021, respectively [11,12]. The following year in June 2022, children between the ages of 6 months and 5 years of age also became eligible for COVID-19 vaccination in the US. Despite the vaccine being approved and available at no cost, national data as of June 2023 show that only 45.6% of children aged 5–17 years are vaccinated with at least one dose and 54.5% remain unvaccinated. The lowest rates of COVID-19 vaccine coverage among children are found in the southern states, including Texas. In Texas, only 1.4% of the 12–17-year-old population are up to date with the COVID-19 vaccine series; 1.1% of 5–11-year-old children and less than 1% of children under the age of 5 are up to date with the pediatric COVID-19 vaccine series [13]. These data clearly reflect Texas parents’ hesitance regarding the COVID-19 vaccine and their lack of intention to vaccinate their children. Key determinants of COVID-19 vaccine hesitancy include mistrust in the safety and efficacy of a COVID-19 vaccine, lower perceived seriousness of the virus and a multitude of sociodemographic factors: political ideology, younger age, women, race, employment status, and lower household income [14,15,16].

Before making any vaccine recommendations to vaccine-hesitant parents, it is important to first understand factors that may influence their concerns regarding the COVID-19 vaccine and their intentions regarding vaccinating their children against COVID-19. During the pandemic, parents have expressed increased doubts about the benefit of vaccines offered by the government and the trustworthiness of healthcare provider recommendations, thinking that doctors would not properly account for their child’s specific medical circumstances [17,18]. Growing evidence also suggests that parental hesitancy towards pediatric COVID-19 vaccination is primarily due to worry about potential long-term ramifications and serious side effects of the relatively new vaccine [19]. In addition to the perceived effectiveness, safety concerns, and trust in the nascent vaccine, parents’ sociodemographic and medical circumstances may play a significant role in their decision-making process regarding their children’s COVID-19 vaccination.

This paper aimed to examine the associations of Texas parents’ sociodemographic factors and medical conditions with having at least one child over 5 years of age who remains unvaccinated against COVID-19. Additionally, we examined factors associated with COVID-19 vaccine intention for children among parents with at least one unvaccinated child.

## 2. Materials and Methods

### 2.1. Study Design and Setting

The COVID-19 Vaccine Hesitancy and Confidence (COVHAC) survey was administered by telephone calls to random phone numbers with Texas area codes from 4 April 2022 until 14 November 2022. The study was approved by the Committee for the Protection of Human Subjects at UTHealth Houston and the UT Tyler Institutional Review Board prior to initiation, and informed verbal consent was obtained at the beginning of each call. We randomly sampled phone numbers across the state to ensure all regions would be proportionately represented. A total of 936,570 phone numbers were dialed, of which 19,502 (2.1%) adults agreed to conduct the survey and answered at least one vaccination question.

### 2.2. Survey Weights

To generalize the findings to the Texas population based on the 2021 American Community Survey 5-year population estimates and to account for the responses gained from the targeted sampling, survey weights were assigned to each respondent. The weights, corresponding to the number of people in the population represented by each respondent, were calculated as (number in subgroup in population)/(number in subgroup in survey). The weights were calculated for age group, sex, race/ethnicity, public health region (PHR), and geographic area; the base weight was the product of each of the individual weights. The final survey weight was standardized by dividing each base weight by the average of the base weights.

### 2.3. Measures

#### 2.3.1. Outcome Variables

To identify households with children, respondents were asked, “Do you have children or any dependents between the ages of 5 and 17 years old?” (Yes/No). The primary outcome was whether a parent had at least one unvaccinated child or all their children vaccinated against COVID-19. Parent respondents were asked, “Have your children/dependents between the ages of 5 and 17 years old received a COVID-19 vaccine?” (Yes/No). The secondary outcome was whether a parent of an unvaccinated child was likely to have their child receive a COVID-19 vaccine in the next three months. Parent respondents were asked, “How likely are you to have your children/dependents between the ages of 5 and 17 years old vaccinated against COVID-19 in the next 3 months?” Response options were: (1) not likely at all; (2) somewhat likely; (3) neither likely nor unlikely; (4) somewhat likely; (5) extremely likely.

#### 2.3.2. Independent Variables

**Figure 1:**
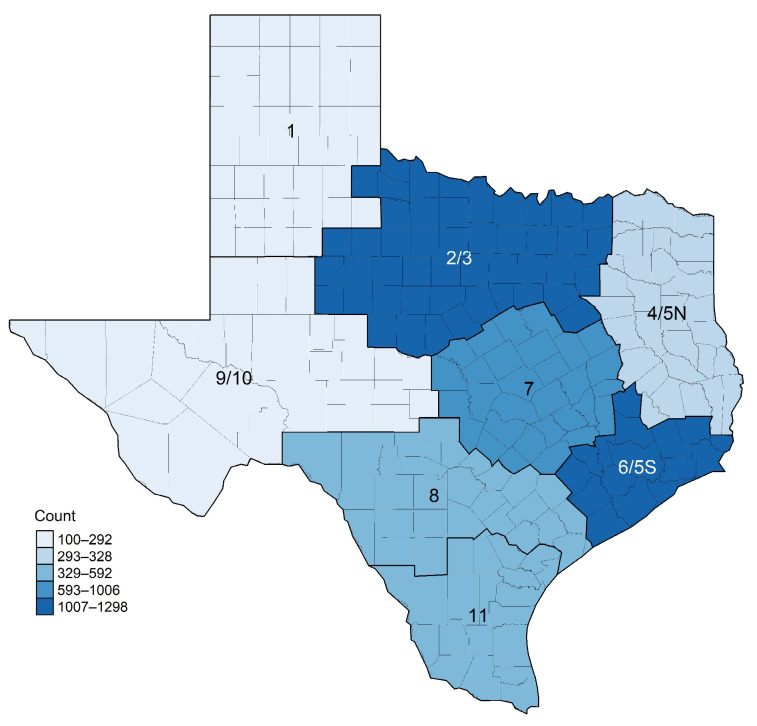
Geographic distribution of survey respondents with children between 5 and 17 years old in public health regions across Texas (*N* = 4879).

Sociodemographic information of parents included the geographic region in Texas (public health region (PHR), see Figure 1), age group, race/ethnicity (White, Hispanic, Asian, Black, Multiracial, and Other), primary language, employment status, and insurance type (recategorized as private, public, uninsured, and other). To assess comorbidities, parents were asked to indicate whether they had any of the listed disease conditions including cancer, chronic disease, COPD, heart disease, obesity, sickle cell, diabetes, immunocompromised, tuberculosis, HIV, hypertension, and other diseases. Additionally, parents were asked to provide the following information about themselves related to the COVID-19 pandemic: history of COVID-19 infection, whether they are concerned about COVID-19-related illness for (a) themselves and (b) others, vaccination status, impact of the pandemic on their work (i.e., loss of work due to the pandemic), and if they struggled to pay monthly bills for basic necessities, such as food, power, and water, due to the COVID-19 pandemic. 

### 2.4. Analysis

Chi-square tests were used to evaluate differences in baseline characteristics by vaccination status. Primary and secondary outcomes were analyzed using multiple logistic regression models that adjusted for each covariate included. Covariates were selected using Lasso regression, where lambda was selected using ten-fold cross-validation to minimize the mean squared error. Standardized survey weights were utilized in the Lasso variable selection and models fit for the primary outcome of whether a respondent is vaccinated, as well as the secondary outcome of whether a respondent is fully vaccinated. Generalized variance-inflation factors were analyzed to rule out multicollinearity, and the Hosmer–Lemeshow test and the area under the receiver operating characteristic curve were used to assess model goodness of fit. Model diagnostics were assessed including the exploration of removing potential influential points. No significant deviations were identified. A *p*-value < 0.05 was considered statistically significant. All analyses were conducted using R version 4.3.0 (R Foundation for Statistical Computing, Vienna, Austria).

## 3. Results

Table 1 presents the unweighted baseline characteristics of respondents (*N* = 4879) with children over age 5 by child vaccination status. More than half of the respondents were females (52.2%) and lived in PHR 6/5S or PHR 2/3 (52.9%). Most were aged 18–49 years (74.3%), White or Hispanic (79.3%), and spoke English (81.7%). In this sample, 53.2% had all their children vaccinated, while 46.8% had at least one unvaccinated child. Among parents with unvaccinated children, 43.9% were 18–39 years old, and 28.8% lived in PHR 2/3, while 44.6% of parents with vaccinated children were 40–49 years old and 26.6% lived in PHR 6/5S. Among parents with unvaccinated children, 49.8% were White, 31.8% Hispanic, and 1.2% Asian; the majority (86.8%) spoke English. Among parents with vaccinated children, 40.5% were Hispanic, 36.9% White, and 2.2% multiracial. Parents with an unvaccinated child were more likely to work in person (62.4% versus 56.4% of those with all children vaccinated). Parents with unvaccinated children were more likely to not be concerned about COVID-19-related illness for themselves and others (42.7% versus 19.3% of those with all children vaccinated). Parents with unvaccinated children were more likely to have had COVID-19 infection without medical care (38.0% versus 29.1% of those with all vaccinated children) and parents of vaccinated children were more likely to have no history of COVID-19 infection (47.1% versus 35.5% of those with unvaccinated children). Parents of unvaccinated children were more likely to be unvaccinated for COVID-19 themselves (54.5% versus 4.5% of those with all vaccinated children), whereas parents of vaccinated children were more likely to be vaccine-boosted (65.3% versus 13.0% of those with unvaccinated children). In both groups, less than 30% expressed that they struggled to pay for basic necessities due to the pandemic. The proportion of parents having the following medical conditions significantly differed by child vaccination status: chronic disease, obesity, diabetes, smoking status, and hypertension.

The Lasso-selected variables included in the adjusted logistic regression analysis (Table 2) were PHR, age group, race/ethnicity, language, insurance type, concern for illness, COVID-19 history, parent vaccination status, struggle to pay for basic necessities due to the pandemic, and the following medical conditions: heart disease, obesity, diabetes, and tuberculosis. Except for PHR 1 and 4/5N, parents in all PHRs had lower odds of having unvaccinated children compared to those living in PHR 6/5S (*p* < 0.001). Parents who were 40–49 years (aOR = 0.44, 95% CI: 0.33–0.59) and 50–64 years (aOR = 0.46, 95% CI: 0.37–0.58) had lower odds of having unvaccinated children, compared to younger (18–39 years) parents. Compared to White parents, Asian (aOR = 0.50, 95% CI: 0.29–0.84) and Black (aOR = 0.69, 95% CI: 0.52–0.91) parents had lower odds of having unvaccinated children, whereas multiracial parents had higher odds (OR = 1.18, 95% CI: 0.62–2.24). Compared to English-speaking parents, Spanish-speaking parents had lower odds of having unvaccinated children (aOR = 0.57, 95% CI: 0.43–0.77). Regarding medical conditions, parents who had heart disease (aOR = 0.41, 95% CI: 0.27–0.63), diabetes (aOR = 0.61, 95% CI: 0.45–0.82), and tuberculosis (aOR = 0.16, 95% CI: 0.03–0.81) had lower odds of having unvaccinated children, while obesity was not a significant factor. Parents showing concerns about COVID-19-related illness for themselves had lower odds of having unvaccinated children (aOR = 0.63, 95% CI: 0.51–0.78). However, being concerned about others’ COVID-19-related illnesses was not associated with the outcome. Compared to COVID-19 vaccine-boosted parents, unvaccinated parents (aOR = 28.60, 95% CI: 21.70–38.00) had greater odds of unvaccinated children. Lastly, parents who struggled to pay for basic necessities due to the pandemic were more likely to have unvaccinated children (aOR = 1.46, 95% CI: 1.19–1.80). 

Table 3 shows the association of parents’ sociodemographic factors and medical conditions with the intention to vaccinate children against COVID-19 in the next three months among parents who have at least one unvaccinated child over 5 years of age. Compared to White parents, Asian (aOR = 6.54, 95% CI: 2.15–19.40), Black (aOR = 3.45, 95% CI: 2.01–5.89), and Hispanic (aOR = 2.91, 95% CI:1.84–4.64) parents had higher odds of intending to vaccinate their children. Parents who spoke Spanish (aOR = 2.81, 95% CI: 1.69–4.70) and other languages (aOR = 2.56, 95% CI: 0.99–6.27) had higher odds of intending to vaccinate their children compared to English-speaking parents. Parents who worked had lower odds of intending to vaccinate their children compared to those who did not work (aOR = 0.61; 95% CI: 0.40–0.92). Parents with chronic disease had higher odds of intending to vaccinate their children (aOR = 3.07, 95% CI: 1.29–7.05); however, other medical conditions, including cancer, COPD, obesity, and hypertension, and a history of COVID-19 infection were not associated with parents’ vaccination intention for their children. Parents concerned about COVID-19-related illness for themselves (aOR = 1.69, 95% CI: 1.17–2.46) and others (aOR = 2.03, 95% CI: 1.37–3.04) had higher odds of intending to vaccinate their children in the next three months. Regarding parents’ own COVID-19 vaccination status, both unvaccinated parents (aOR = 0.11, 95% CI: 0.07–0.19) and partially vaccinated parents (aOR = 0.57, 95% CI: 0.27–1.15) had lower odds of intending to vaccinate their children compared to vaccine-boosted parents. Parents’ PHR, age, and insurance status were not significantly associated with children’s likelihood of receiving the COVID-19 vaccine in the next three months.

## 4. Discussion

This study examined the associations of parents’ sociodemographic factors and medical conditions with children’s receipt of the COVID-19 vaccine and their likelihood of vaccination, using data from the COVID-19 Vaccine Hesitancy and Confidence (COVHAC) survey. In this sample, parental hesitancy towards the COVID-19 vaccine for their children was prevalent and parents’ intention to vaccinate their children varied by a number of parental factors. Below we highlight the main findings and discuss their implications.

Race is a key contributing demographic factor behind COVID-19 vaccine hesitancy. While previous systematic reviews have reported lower COVID-19 vaccine intention for children among Asian and Black parents compared to White parents [20,21], we observed an opposite trend in this sample. We found that compared to White parents, Asian and Black parents in Texas had higher vaccine intention for their children, resulting in a lower likelihood of having unvaccinated children. Similarly, non-English-speaking parents had lower odds of having unvaccinated children and higher odds of intention to vaccinate their children, compared to their English-speaking counterparts. These findings corroborate greater openness to COVID-19 vaccination and vaccine acceptance among Hispanic, Black, and Asian parents compared to White parents in this sample. As in other parts of the US, Hispanics and Blacks in Texas are disproportionately affected by COVID-19 [22,23,24]. Their elevated risk of COVID-19 infection is largely due to systemic and structural inequities contributing to a higher prevalence of precarious conditions [25]. For example, the types of jobs held and the distribution of risks within the workplace during the pandemic are patterned by race and ethnicity [26,27]. Substantial percentages of frontline workers from marginalized racial/ethnic groups likely resulted in greater exposure to infection than other Americans who worked from home during the pandemic [28]. Moreover, racial/ethnic minorities tend to live in multigenerational households, which is detrimental to the fight against the spread of the virus. Multigenerational living increases vulnerability to unnoticed infections through asymptomatic children who could swiftly infect their elders [29,30]. Parents of racial/ethnic minorities may be more inclined to vaccinate their children against COVID-19 due to heightened awareness of their circumstances and susceptibility to the infection. 

The PHR of parents was also a significant sociodemographic factor and we confirmed geographic variations in COVID-19 vaccine uptake among children over age 5. Specifically, we observed that parents from PHR 1 and 4/5N were more likely to have un-vaccinated children compared to those from PHR 6/5S, which is urban and the second most populated region in Texas [31]. PHR 1 and 4/5N are substantially rural, have higher proportions of English-speaking residents, and are some of the least densely populated regions in the state [31,32]. County-level data from the Centers for Disease Control and Prevention have shown that COVID-19 vaccination rates are significantly lower in rural than in urban counties and that vaccination rates decline monotonically with rurality [33]. In addition, the work status of parents significantly influenced children’s uptake of the COVID-19 vaccine. Parents who were employed were less likely to vaccinate their children against COVID-19 compared to those who were not employed. However, other studies have found that unemployed parents were more likely to reject the COVID-19 vaccine for their children or parents’ employment status was not associated with vaccination intention for their children [34,35]. While parents working full time may face time constraints or logistical challenges in taking their children to the clinic for routine childhood vaccines, this may not apply to the COVID-19 vaccines as long-hour, walk-in mass vaccination events were organized during the pandemic. COVID-19 vaccines are readily available and do not pose any logistical challenges for working parents. Therefore, the reluctance of working parents to vaccinate their children may be attributed to their persistent negative attitudes towards vaccines or the influence of other vaccine-hesitant parent colleagues, rather than logistical constraints. 

As expected, children’s receipt of the COVID-19 vaccine was strongly associated with parents’ own COVID-19 vaccination status and their level of concern for COVID-19-related illness. We found that children’s likelihood of receiving the vaccine was much lower among unvaccinated and partially vaccinated parents, compared to parents who had received a booster dose. These findings align with previous studies that identified parents’ vaccination status, willingness to be vaccinated, and vaccine hesitancy as prominent factors independently associated with children’s likelihood of receiving a COVID-19 vaccine [36,37,38]. One of these studies noted that only 10% of parents who received the COVID-19 vaccine were reluctant to vaccinate their children [38]. The “wait-and-see” attitude is a common sentiment among parents regarding any new vaccines. It is possible that vaccinated parents in our study may have preferred to adopt the wait-and-see approach first and decided to vaccinate their children only after confirming vaccine safety and the absence of side effects from the vaccine on themselves. Our study also revealed that parents who had concerns for themselves and others about COVID-19-related illness were more likely to vaccinate their children. A potential explanation for these findings is that individuals with altruistic beliefs and a strong sense of collective responsibility may have stronger perceptions of vaccines as highly effective in protecting others. Consequently, acceptance of the COVID-19 vaccine for children may be driven by parents who exhibit these attributes [36,39]. 

In our study, a participant indicating that they struggle to pay for basic necessities due to the pandemic was used as an indicator of household financial insecurity and low socioeconomic status. Individuals with low socioeconomic status, regardless of employment, have become more economically vulnerable across a range of outcomes during the pandemic, including the ability to pay for essential expenses, and perceived economic hardship may be greater for these people [40,41]. Our study adds to the existing body of research by demonstrating how household financial insecurity is associated with higher levels of vaccine hesitancy and ultimately hinders parents from vaccinating children against COVID-19 [10,42,43]. While the COVID-19 vaccine was free and readily accessible after the initial rollout, we found that parents who struggled to pay for basic necessities due to the pandemic were more likely to have unvaccinated children, compared to those who did not experience financial barriers. A recent study also reported that parents with lower education and income levels were less likely to intend to vaccinate their children against COVID-19 [44]. These results, along with ours, are not surprising. Unlike parents of high socioeconomic status, parents of low socioeconomic status may not have computer access or internet availability to acquire information and deploy technology for healthcare into the home environment [45]. Consequently, they may lack updated information about the severity of COVID-19 infection and benefits of vaccination [46], which could heighten resistance to vaccinating their children. At the same time, however, they may not encounter social media that might include vaccine misinformation. Another barrier could be the incapability to make online reservations for immunizations, a requirement enforced by many vaccine programs and initiatives [47]. In addition, without proper knowledge that the COVID-19 vaccine is provided at no cost, parents with low income may fear unaffordability of the vaccine and subsequently forgo vaccination for their children. 

An individual’s health status significantly influences their willingness to receive vaccinations [48,49], however, there is limited research on the relationship between parents’ medical history and their children’s COVID-19 vaccine uptake. A few studies have explored the relationship between parents’ mental illness and intention to vaccinate their children against COVID-19. For example, Latin American parents with depression demonstrated a higher intention to vaccinate their children [50], whereas US mothers with posttraumatic stress disorder had significantly less confidence in the vaccine [51]. Among patients with chronic conditions, the odds of contracting the virus are 2.5–3.9 times higher [52], and once infected, they are more likely to develop serious complications that can lead to mortality [53]. The mortality rate of unvaccinated patients exceeds that of fully vaccinated or boosted patients [54], but uninformed patients may perceive that their primary disease would aggravate after COVID-19 vaccination and worry about bad vaccine reaction. Depending on disease severity and their belief that the disease is hereditary, patients who have children may decide against the vaccine for not only themselves but also for their children. In contrast, other studies reported that patients with chronic conditions have a higher perceived risk of and susceptibility to COVID-19 and indicated a willingness to receive a COVID-19 vaccine [55,56,57]. The fear of becoming more easily infected with COVID-19 likely contributed to increased vaccine acceptance in this population [57]. In this regard, our results suggest that a parent with chronic illnesses may have a positive assessment of the usefulness of the COVID-19 vaccine and willingness to vaccinate their children [50]. We note that the non-significant associations of parents’ obesity with vaccination intention and having unvaccinated children in this sample may have been impacted by the fact that many overweight or obese individuals often do not self-identify as such [58]. 

Limitations of the study include the use of a non-validated survey, a low response rate to the survey, and restriction of the analysis to parents of children over the age of 5, as survey administration ended shortly after approval of COVID-19 vaccination for children under the age of 5. Despite the large sample size, the low response rate may be subject to non-response bias and limit the generalizability of the findings. The absence of questions regarding parents’ religion and political views in the survey is also a limitation. We advise caution in inferring causality between the study variables given the cross-sectional nature of the study. Implementing a longitudinal design could help understand changes in vaccination over time and establish causal relationships. This study has several strengths. First, we conducted a statewide survey representative across all public health regions in Texas. Second, we used the Lasso penalization method which is effective for controlling multicollinearity in regression models with many covariates. Finally, to the best of our knowledge, this is one of the only studies to consider parents’ medical conditions as a potential factor influencing children’s COVID-19 vaccine uptake. To delve deeper into our quantitative findings and add to the sparse literature, future research should qualitatively explore how parents with chronic diseases perceive pediatric COVID-19 vaccination and make vaccination decisions for their children. Future research avenues also include examining parents’ vaccine decisions in diverse geographic regions to enhance the understanding of regional variations in vaccine hesitancy.

## 5. Conclusions

The findings of this study show that various sociodemographic factors and medical conditions of parents influence the likelihood of their children’s COVID-19 vaccine uptake in Texas. Specifically, parents’ race/ethnicity, vaccination status, concerns about contracting the virus and its long-term consequences for themselves and others, household financial insecurity, and chronic diseases were associated with the intention to vaccinate their children against COVID-19. As children comprise a substantial proportion of the population in the United States, their vaccination is critical to reducing the rate of infection. It will be important to raise awareness about the safety, efficacy, and importance of pediatric COVID-19 vaccination, tailored to parents’ sociodemographic and medical circumstances.

## Figures and Tables

**Table 1:** Baseline characteristics for community respondents by child vaccination status for children over 5 years of age.

Respondents, *N* (%) (Unless Otherwise Indicated)					
	Missing % ^‡^	Overall	At Least One Unvaccinated Child over 5	All Children over 5 Vaccinated	*p*-Value *^,†^
Total Number of Respondents		4879	2283 (46.8)	2596 (53.2)	
PHR	1.7				**<0.001**
6/5S		1238 (25.8)	561 (24.9)	677 (26.6)	
1		100 (2.1)	68 (3.0)	32 (1.3)	
11		329 (6.9)	108 (4.8)	221 (8.7)	
2/3		1298 (27.1)	648 (28.8)	650 (25.6)	
4/5N		325 (6.8)	211 (9.4)	114 (4.5)	
7		660 (13.8)	281 (12.5)	379 (14.9)	
8		575 (12.0)	255 (11.3)	320 (12.6)	
9/10		270 (5.6)	119 (5.3)	151 (5.9)	
Age Group	5.7				**<0.001**
Age 18–39		1569 (34.1)	946 (43.9)	623 (25.4)	
Age 40–49		1852 (40.2)	761 (35.3)	1091 (44.6)	
Age 50–64		1006 (21.9)	383 (17.8)	623 (25.4)	
Age 65+		176 (3.8)	65 (3.0)	111 (4.5)	
Sex/Gender	0.3				**<0.001**
Female		2541 (52.2)	1095 (48.1)	1446 (55.8)	
Male		2262 (46.5)	1147 (50.4)	1115 (43.0)	
Other		63 (1.3)	33 (1.5)	30 (1.2)	
Race/Ethnicity	1.8				**<0.001**
White		2055 (42.9)	1112 (49.8)	943 (36.9)	
Asian		163 (3.4)	27 (1.2)	136 (5.3)	
Black		501 (10.5)	204 (9.1)	297 (11.6)	
Hispanic		1745 (36.4)	709 (31.8)	1036 (40.5)	
Multiracial		115 (2.4)	60 (2.7)	55 (2.2)	
Other		211 (4.4)	120 (5.4)	91 (3.6)	
Language	0.2				**<0.001**
English		3979 (81.7)	1979 (86.8)	2000 (77.2)	
Other		166 (3.4)	57 (2.5)	109 (4.2)	
Spanish		726 (14.9)	245 (10.7)	481 (18.6)	
Work	0.4	3870 (79.6)	1848 (81.3)	2022 (78.2)	**0.007**
Work Situation	21.4				**<0.001**
Remote		506 (13.2)	199 (10.9)	307 (15.3)	
In person		2273 (59.2)	1142 (62.4)	1131 (56.4)	
Hybrid		1058 (27.6)	490 (26.8)	568 (28.3)	
No Work—Reason	80.1				0.319
Temporary lay off		50 (5.1)	23 (5.5)	27 (4.9)	
Voluntary separation		45 (4.6)	16 (3.8)	29 (5.2)	
Permanently laid off		33 (3.4)	14 (3.3)	19 (3.4)	
Retired		184 (18.9)	67 (16.0)	117 (21.1)	
Student		54 (5.6)	23 (5.5)	31 (5.6)	
Other		606 (62.3)	275 (65.8)	331 (59.7)	
No Work—COVID Related	98.1	37 (39.4)	19 (50.0)	18 (32.1)	0.128
Insurance ^§^	5.8				**<0.001**
Other		293 (6.4)	150 (7.0)	143 (5.8)	
Private (+)		2864 (62.3)	1280 (59.6)	1584 (64.7)	
Public		798 (17.4)	374 (17.4)	424 (17.3)	
Uninsured		639 (13.9)	343 (16.0)	296 (12.1)	
Cancer	0.0	133 (2.7)	54 (2.4)	79 (3.0)	0.174
Chronic Disease	0.0	169 (3.5)	62 (2.7)	107 (4.1)	**0.009**
COPD	0.0	62 (1.3)	30 (1.3)	32 (1.2)	0.899
Heart Disease	0.0	189 (3.9)	85 (3.7)	104 (4.0)	0.664
Obesity	0.0	641 (13.1)	253 (11.1)	388 (14.9)	**<0.001**
Sickle Cell	0.0	30 (0.6)	15 (0.7)	15 (0.6)	0.864
Diabetes	0.0	420 (8.6)	140 (6.1)	280 (10.8)	**<0.001**
Immunocompromised	0.0	43 (0.9)	21 (0.9)	22 (0.8)	0.906
Smoker	0.0	435 (8.9)	261 (11.4)	174 (6.7)	**<0.001**
Tuberculosis	0.0	22 (0.5)	10 (0.4)	12 (0.5)	1.00
HIV	0.0	17 (0.3)	10 (0.4)	7 (0.3)	0.451
Hypertension	0.0	599 (12.3)	225 (9.9)	374 (14.4)	**<0.001**
Other Disease	4.0	316 (6.7)	130 (5.9)	186 (7.4)	**0.047**
Concern for Illness: Personal	0.3				**<0.001**
Not concerned at all		2213 (45.5)	1400 (61.5)	813 (31.5)	
Somewhat not concerned		585 (12.0)	240 (10.5)	345 (13.4)	
Neither		79 (1.6)	43 (1.9)	36 (1.4)	
Somewhat concerned		1459 (30.0)	455 (20.0)	1004 (38.9)	
Extremely concerned		526 (10.8)	140 (6.1)	386 (14.9)	
Concern for Illness: Others	0.4				**<0.001**
Not concerned at all		1471 (30.3)	972 (42.7)	499 (19.3)	
Somewhat not concerned		445 (9.2)	216 (9.5)	229 (8.9)	
Neither		70 (1.4)	34 (1.5)	36 (1.4)	
Somewhat concerned		1729 (35.6)	691 (30.4)	1038 (40.2)	
Extremely concerned		1143 (23.5)	363 (15.9)	780 (30.2)	
COVID History	2.9				**<0.001**
No		1976 (41.7)	787 (35.5)	1189 (47.1)	
Yes, medical care		1185 (25.0)	586 (26.5)	599 (23.8)	
Yes, no medical care		1576 (33.3)	842 (38.0)	734 (29.1)	
Parent Vaccination Status	0.5				**<0.001**
Boosted		1981 (40.8)	296 (13.0)	1685 (65.3)	
Non-boosted		1353 (27.9)	634 (27.9)	719 (27.8)	
Not fully vaccinated		166 (3.4)	103 (4.5)	63 (2.4)	
Unvaccinated		1354 (27.9)	1239 (54.5)	115 (4.5)	
**Struggle to Pay**	0.2	1304 (26.8)	675 (29.7)	629 (24.3)	**<0.001**

^‡^ Missing percentage is taken out of overall total. *N* = number of respondents; PHR = public health region. * *p*-values represent significance level of the chi-square test of independence between randomized groups for binary and categorical variables, or the one-way analysis of variance (ANOVA) between randomized groups for continuous variables. ^†^ Statistically significant *p*-values (<0.05) are shown in bold text. ^§^ Insurance category of Private (+) includes all private insurance options, Public includes Medicare, Medicaid, Medigap, CHIP, Military, Indian, State-Sponsored, and Affordable Care Act options, and Other includes those who have insurance but do not know which option and those with options not listed. Note that questions with higher missing percentages were only asked if appropriate, so not every respondent had the option to answer. For instance, only respondents who claimed to not work answered No Work—Reason and No Work—COVID Related.

**Table 2:** Adjusted ^‡^ odds ratios for having at least one unvaccinated child over 5 years of age among community respondents.

	With All Variables			With Lasso-Selected Variables		
	Odds Ratio	95% CI	*p*-Value *^,†^	Odds Ratio	95% CI	*p*-Value *^,†^
PHR			**<0.001**			**<0.001**
6/5S	-	-		-	-	
1	1.47	0.88, 2.50		1.51	0.91, 2.54	
11	0.37	0.26, 0.51		0.39	0.28, 0.55	
2/3	0.92	0.73, 1.16		0.94	0.75, 1.18	
4/5N	1.09	0.69, 1.72		1.12	0.72, 1.76	
7	0.78	0.57, 1.07		0.80	0.58, 1.09	
8	0.53	0.38, 0.73		0.54	0.39, 0.75	
9/10	0.84	0.56, 1.24		0.84	0.57, 1.25	
Age Group			**<0.001**			**<0.001**
Age 18–39	-	-		-	-	
Age 40–49	0.43	0.32, 0.58		0.44	0.33, 0.59	
Age 50–64	0.44	0.35, 0.55		0.46	0.37, 0.58	
Age 65+	1.09	0.82, 1.45		1.11	0.85, 1.45	
Race/Ethnicity			**0.004**			**0.008**
White	-	-		-	-	
Asian	0.50	0.29, 0.84		0.50	0.29, 0.84	
Black	0.66	0.49, 0.87		0.69	0.52, 0.91	
Hispanic	1.01	0.80, 1.28		1.00	0.80, 1.27	
Multiracial	1.18	0.62, 2.26		1.18	0.62, 2.24	
Other	0.26	0.01, 1.59		0.26	0.01, 1.62	
Language			**<0.001**			**<0.001**
English	-	-		-	-	
Other	0.89	0.51, 1.52		0.90	0.52, 1.55	
Spanish	0.57	0.43, 0.77		0.57	0.43, 0.77	
Work	1.13	0.90, 1.42	0.30			
Insurance ^§^			**0.04**			**0.04**
Other	-	-		-	-	
Private (+)	0.61	0.41, 0.90		0.61	0.41, 0.90	
Public	0.72	0.47, 1.10		0.70	0.46, 1.07	
Uninsured	0.57	0.37, 0.89		0.57	0.37, 0.88	
Cancer	1.00	0.60, 1.65	>0.99			
Chronic Disease	0.88	0.54, 1.42	0.61			
COPD	0.67	0.29, 1.50	0.33			
Heart Disease	0.41	0.26, 0.65	**<0.001**	0.41	0.27, 0.63	**<0.001**
Obesity	0.82	0.62, 1.08	0.16	0.88	0.67, 1.15	0.35
Sickle Cell	0.62	0.22, 1.67	0.34			
Diabetes	0.60	0.44, 0.82	**0.001**	0.61	0.45, 0.82	**0.001**
Immunocompromised	1.54	0.53, 4.16	0.41			
Smoker	1.00	0.72, 1.40	>0.99			
Tuberculosis	0.16	0.03, 0.80	**0.027**	0.16	0.03, 0.81	**0.03**
HIV	0.55	0.08, 3.11	0.50			
Hypertension	1.48	1.13, 1.95	**0.005**			
Other Disease	1.05	0.75, 1.46	0.77			
Concern for Illness: Personal	0.62	0.50, 0.76	**<0.001**	0.63	0.51, 0.78	**<0.001**
Concern for Illness: Others	0.95	0.77, 1.17	0.64	0.96	0.78, 1.18	0.70
COVID History			**0.025**			**0.03**
No	-	-		-	-	
Yes, medical care	1.35	1.08, 1.69		1.35	1.08, 1.68	
Yes, no medical care	1.06	0.86, 1.30		1.06	0.87, 1.31	
Parent Vaccination Status			**<0.001**			**<0.001**
Boosted	-	-		-	-	
Non-boosted	4.23	3.46, 5.18		4.11	3.37, 5.02	
Not fully vaccinated	11.50	7.37, 18.50		11.20	7.19, 17.90	
Unvaccinated	29.40	22.30, 39.10		28.60	21.70, 38.00	
Struggle to Pay	1.49	1.21, 1.84	**<0.001**	1.46	1.19, 1.80	**<0.001**

CI = confidence interval; PHR = public health region. ^‡^ Adjusted for each covariate shown, in addition to all others presented in the table. * *p*-values represent the significance level of the likelihood ratio test for each factor within the model. ^†^ Statistically significant *p*-values (<0.05) are shown in bold text. ^§^ Insurance category of Private (+) includes all private insurance options, Public includes Medicare, Medicaid, Medigap, CHIP, Military, Indian, State-Sponsored, and Affordable Care Act options, and Other includes those who have insurance but do not know which option and those with options not listed.

**Table 3:** Adjusted ^‡^ odds ratios for having at least one unvaccinated child over 5 years of age likely to receive vaccine in next 3 months among community respondents.

Variable	Odds Ratio	95% CI	*p*-Value *^,†^
PHR			0.31
6/5S	-	-	
1	1.69	0.52, 4.72	
11	1.13	0.49, 2.48	
2/3	1.28	0.83, 1.98	
4/5N	0.70	0.29, 1.52	
7	0.94	0.50, 1.71	
8	1.35	0.76, 2.37	
9/10	0.52	0.20, 1.20	
Age Group			0.37
Age 18–39	-	-	
Age 40–49	0.73	0.49, 1.07	
Age 50–64	0.87	0.53, 1.41	
Age 65+	0.62	0.25, 1.46	
Race/Ethnicity			**<0.001**
White	-	-	
Asian	6.54	2.15, 19.40	
Black	3.45	2.01, 5.89	
Hispanic	2.91	1.84, 4.64	
Multiracial	0.68	0.10, 2.49	
Other	0.97	0.30, 2.56	
Language			**<0.001**
English	-	-	
Other	2.56	0.99, 6.27	
Spanish	2.81	1.69, 4.70	
Work	0.61	0.40, 0.92	**0.019**
Insurance ^§^			0.57
Other	-	-	
Private (+)	0.96	0.49, 2.00	
Public	0.83	0.39, 1.83	
Uninsured	1.23	0.59, 2.67	
Cancer	2.17	0.82, 5.31	0.12
Chronic Disease	3.07	1.29, 7.05	**0.012**
COPD	1.41	0.39, 4.50	0.58
Obesity	1.32	0.79, 2.17	0.29
Hypertension	1.17	0.67, 1.99	0.57
Concern for Illness: Personal	1.69	1.17, 2.46	**0.006**
Concern for Illness: Others	2.03	1.37, 3.04	**<0.001**
COVID History			0.19
No	-	-	
Yes, medical care	0.75	0.49, 1.15	
Yes, no medical care	0.71	0.48, 1.05	
Parent Vaccination Status			**<0.001**
Boosted	-	-	
Non-boosted	0.56	0.37, 0.84	
Not fully vaccinated	0.57	0.27, 1.15	
Unvaccinated	0.11	0.07, 0.19	

CI = confidence interval; PHR = public health region. ^‡^ Adjusted for each covariate shown, in addition to all others presented in the table. * *p*-values represent significance level of the likelihood ratio test for each factor within the model. ^†^ Statistically significant *p*-values (<0.05) are shown in bold text. ^§^ Insurance category of Private (+) includes all private insurance options, Public includes Medicare, Medicaid, Medigap, CHIP, Military, Indian, State-Sponsored, and Affordable Care Act options, and Other includes those who have insurance but do not know which option and those with options not listed.

## Data Availability

The researchers have a contractual agreement with the owner of the data that restricts data sharing with third parties.

## References

[1] Children and COVID-19: State-Level Data Report. https://www.aap.org/en/pages/2019-novel-coronavirus-covid-19-infections/children-and-covid-19-state-level-data-report/.

[2] Messiah S.E., Francis J., Weerakoon S., Mathew M.S., Shaikh S., Veeraswamy A., Lozano A., He W., Xie L., Polavarapu D. (2023). Persistent symptoms and conditions among children and adolescents hospitalised with COVID-19 illness: A qualitative study. BMJ Open.

[3] Borel M., Xie L., Kapera O., Mihalcea A., Kahn J., Messiah S.E. (2022). Long-term physical, mental and social health effects of COVID-19 in the pediatric population: A scoping review. World J. Pediatr..

[4] Flaxman S., Whittaker C., Semenova E., Rashid T., Parks R.M., Blenkinsop A., Unwin H.J., Mishra S., Bhatt S., Gurdasani D. (2023). Assessment of COVID-19 as the underlying cause of death among children and young people aged 0 to 19 years in the US. JAMA Netw. Open.

[5] Opel D.J., Diekema D.S., Ross L.F. (2021). Should we mandate a COVID-19 vaccine for children?. JAMA Pediatr..

[6] Alhazza S.F., Altalhi A.M., Alamri K.M., Alenazi S.S., Alqarni B.A., Almohaya A.M. (2022). Parents’ hesitancy to vaccinate their children against COVID-19, a country-wide survey. Front. Public Health.

[7] Kelvin A.A., Halperin S. (2020). COVID-19 in children: The link in the transmission chain. Lancet Infect. Dis..

[8] Ruiz J.B., Bell R.A. (2022). Parental COVID-19 vaccine hesitancy in the United States. Public Health Rep..

[9] Eberhardt C.S., Siegrist C.A. (2021). Is there a role for childhood vaccination against COVID-19?. Pediatr. Allergy Immunol..

[10] Fisher C.B., Bragard E., Jaber R., Gray A. (2022). COVID-19 vaccine hesitancy among parents of children under five years in the United States. Vaccines.

[11] FDA Approves First COVID-19 Vaccine. https://www.fda.gov/news-events/press-announcements/fda-approves-first-covid-19-vaccine.

[12] Woodworth K.R., Moulia D., Collins J.P., Hadler S.C., Jones J.M., Reddy S.C., Chamberland M., Campos-Outcalt D., Morgan R.L., Brooks O. (2021). The Advisory Committee on Immunization Practices’ interim recommendation for use of Pfizer-BioNTech COVID-19 vaccine in children aged 5–11 years—United States, November 2021. MMWR Morb. Mortal. Wkly. Rep..

[13] COVID Data Tracker Atlanta, GA: U.S. Department of Health and Human Services, CDC. https://covid.cdc.gov/covid-data-tracker/#vaccination-demographics-maps.

[14] Gerretsen P., Kim J., Caravaggio F., Quilty L., Sanches M., Wells S., Brown E.E., Agic B., Pollock B.G., Graff-Guerrero A. (2021). Individual determinants of COVID-19 vaccine hesitancy. PLoS ONE.

[15] Roy D.N., Hossen M.M., Biswas M., Islam E., Azam M.S. (2022). Prevalence of COVID-19 vaccine hesitancy in students: A global systematic review. F1000Research.

[16] Soares P., Rocha J.V., Moniz M., Gama A., Laires P.A., Pedro A.R., Dias S., Leite A., Nunes C. (2021). Factors associated with COVID-19 vaccine hesitancy. Vaccines.

[17] He K., Mack W.J., Neely M., Lewis L., Anand V. (2022). Parental perspectives on immunizations: Impact of the COVID-19 pandemic on childhood vaccine hesitancy. J. Community Health.

[18] Smith L.E., Amlôt R., Weinman J., Yiend J., Rubin G.J. (2017). A systematic review of factors affecting vaccine uptake in young children. Vaccine.

[19] Suran M. (2022). Why parents still hesitate to vaccinate their children against COVID-19. JAMA.

[20] Galanis P., Vraka I., Fragkou D., Bilali A., Kaitelidou D. (2021). Intention of healthcare workers to accept COVID-19 vaccination and related factors: A systematic review and meta-analysis. Asian Pac. J. Trop. Med..

[21] Galanis P., Vraka I., Siskou O., Konstantakopoulou O., Katsiroumpa A., Kaitelidou D. (2022). Willingness, refusal and influential factors of parents to vaccinate their children against the COVID-19: A systematic review and meta-analysis. Prev. Med..

[22] Adepoju O.E., Ojinnaka C.O. (2021). County-level determinants of COVID-19 testing and cases: Are there racial/ethnic disparities in Texas?. Popul. Health Manag..

[23] Ojinnaka C.O., Adepoju O.E., Burgess A.V., Woodard L. (2021). Factors associated with COVID-related mortality: The case of Texas. J. Racial Ethn. Health Disparities.

[24] Xu A., Loch-Temzelides T., Adiole C., Botton N., Dee S.G., Masiello C.A., Osborn M., Torres M.A., Cohan D.S. (2022). Race and ethnic minority, local pollution, and COVID-19 deaths in Texas. Sci. Rep..

[25] Valier M.R., Elam-Evans L.D., Mu Y., Santibanez T.A., Yankey D., Zhou T., Pingali C., Singleton J.A. (2023). Racial and ethnic differences in COVID-19 vaccination coverage among children and adolescents aged 5–17 years and parental intent to vaccinate their children—National immunization survey–child COVID module, United States, December 2020–September 2022. MMWR Morb. Mort. Wkly. Rep..

[26] Fujishiro K., Ahonen E.Q., de Porras D.G.R., Chen I.C., Benavides F.G. (2021). Sociopolitical values and social institutions: Studying work and health equity through the lens of political economy. SSM Popul. Health.

[27] Haro-Ramos A.Y., Brown T.T., Deardorff J., Aguilera A., Pollack Porter K.M., Rodriguez H.P. (2023). Frontline work and racial disparities in social and economic pandemic stressors during the first COVID-19 surge. Health Serv. Res..

[28] Rho H.J., Brown H., Fremstad S. (2020). A basic demographic profile of workers in frontline industries. Cent. Econ. Policy Res..

[29] Dowd J.B., Andriano L., Brazel D.M., Rotondi V., Block P., Ding X., Liu Y., Mills M.C. (2020). Demographic science aids in understanding the spread and fatality rates of COVID-19. Proc. Natl. Acad. Sci. USA.

[30] Yang W., Shaff J., Shaman J. (2021). Effectiveness of non-pharmaceutical interventions to contain COVID-19: A case study of the 2020 spring pandemic wave in New York City. J. R. Soc. Interface.

[31] Texas Department of State Health Services Center for Health Statistics GIS Team and Map Collection. https://center-for-health-statistics-gis-map-collection-txdshsea.hub.arcgis.com/.

[32] Texas Department of State Health Services Texas Health Data. https://healthdata.dshs.texas.gov/dashboard/surveys-and-profiles/health-facts-profiles/population-profiles.

[33] Sun Y., Monnat S.M. (2022). Rural-urban and within-rural differences in COVID-19 vaccination rates. J. Rural Health.

[34] Bell S., Clarke R., Mounier-Jack S., Walker J.L., Paterson P. (2020). Parents’ and guardians’ views on the acceptability of a future COVID-19 vaccine: A multi-methods study in England. Vaccine.

[35] Humble R.M., Sell H., Dubé E., MacDonald N.E., Robinson J., Driedger S.M., Sadarangani M., Meyer S.B., Wilson S., Benzies K.M. (2021). Canadian parents’ perceptions of COVID-19 vaccination and intention to vaccinate their children: Results from a cross-sectional national survey. Vaccine.

[36] Krakowczyk J.B., Bäuerle A., Pape L., Kaup T., Nulle L., Teufel M., Skoda E.M. (2022). COVID-19 vaccine for children: Vaccination willingness of parents and its associated factors—A network analysis. Vaccines.

[37] Szilagyi P.G., Shah M.D., Delgado J.R., Thomas K., Vizueta N., Cui Y., Vangala S., Shetgiri R., Kapteyn A. (2021). Parents’ intentions and perceptions about COVID-19 vaccination for their children: Results from a national survey. Pediatrics.

[38] Nguyen K.H., Nguyen K., Geddes M., Allen J.D., Corlin L. (2022). Trends in adolescent COVID-19 vaccination receipt and parental intent to vaccinate their adolescent children, United States, July to October, 2021. Ann. Med..

[39] Burke P.F., Masters D., Massey G. (2021). Enablers and barriers to COVID-19 vaccine uptake: An international study of perceptions and intentions. Vaccine.

[40] Perry B.L., Aronson B., Pescosolido B.A. (2021). Pandemic precarity: COVID-19 is exposing and exacerbating inequalities in the American heartland. Proc. Natl. Acad. Sci. USA.

[41] Hibel L.C., Boyer C.J., Buhler-Wassmann A.C., Shaw B.J. (2021). The psychological and economic toll of the COVID-19 pandemic on Latina mothers in primarily low-income essential worker families. Traumatology.

[42] Shmueli L. (2023). Parents’ intention to vaccinate their 5-to 11-year-old children with the COVID-19 vaccine: Rates, predictors and the role of incentives. BMC Public Health.

[43] Yılmaz M., Sahin M.K. (2021). Parents’ willingness and attitudes concerning the COVID-19 vaccine: A cross-sectional study. Int. J. Clin. Pract..

[44] Hetherington E., Edwards S.A., MacDonald S.E., Racine N., Madigan S., McDonald S., Tough S. (2021). SARS-CoV-2 vaccination intentions among mothers of children aged 9 to 12 years: A survey of the All Our Families cohort. CMAJ Open.

[45] Livingood W.C., Bautista M.A., Smotherman C., Azueta D., Coleman J., Grewal R., Stewart E., Orlando L.A., Scuderi C. (2022). Comparative study of different SES neighborhood clinics for health literacy and internet access. Digit. Health.

[46] Mekonnen C.K., Demissie N.G., Beko Z.W., Ferede Y.M., Abate H.K. (2022). Intent to get vaccinated against COVID-19 pandemic and its associated factors among adults with a chronic medical condition. Int. J. Afr. Nurs. Sci..

[47] Lee S., Kim S. (2023). Public’s Experience with an Online Reservation System for Residual COVID-19 Vaccines and the Potential for Increasing the Actual Vaccination Rate. Vaccines.

[48] Ying C.Q., Lin X.Q., Lv L., Chen Y., Jiang J.J., Zhang Y., Tung T.H., Zhu J.S. (2022). Intentions of patients with hypertension to receive a booster dose of the COVID-19 vaccine: A cross-sectional survey in Taizhou, China. Vaccines.

[49] Li H., Ping F., Li X., Wang Z., Xiao J., Jiang H., Xue Y., Quan J., Yao H., Zheng X. (2023). COVID-19 vaccine coverage, safety, and perceptions among patients with diabetes mellitus in China: A cross-sectional study. Front. Endocrinol..

[50] Urrunaga-Pastor D., Herrera-Añazco P., Uyen-Cateriano A., Toro-Huamanchumo C.J., Rodriguez-Morales A.J., Hernandez A.V., Benites-Zapata V.A., Bendezu-Quispe G. (2021). Prevalence and factors associated with parents’ non-intention to vaccinate their children and adolescents against COVID-19 in Latin America and the Caribbean. Vaccines.

[51] Milan S., Dáu A.L.B. (2021). The role of trauma in mothers’ COVID-19 vaccine beliefs and intentions. J. Pedatr. Psychol..

[52] Wang B., Li R., Lu Z., Huang Y. (2020). Does comorbidity increase the risk of patients with COVID-19: Evidence from meta-analysis. Aging.

[53] Richardson S., Hirsch J.S., Narasimhan M., Crawford J.M., McGinn T., Davidson K.W., Barnaby D.P., Becker L.B., Chelico J.D., Cohen S.L. (2020). Presenting characteristics, comorbidities, and outcomes among 5700 patients hospitalized with COVID-19 in the New York City area. JAMA.

[54] Zhao H., Ye W., Yu X., Shi Y., Sheng J. (2023). Omicron COVID-19 variant outcomes and vaccination in non-severe and non-critical patients at admission. Front. Public Health.

[55] Laires P.A., Dias S., Gama A., Moniz M., Pedro A.R., Soares P., Aguiar P., Nunes C. (2021). The association between chronic disease and serious COVID-19 outcomes and its influence on risk perception: Survey study and database analysis. JMIR Public Health Surveill..

[56] Opsasnick L.A., Curtis L.M., Kwasny M.J., O’Conor R., Wismer G.A., Benavente J.Y., Lovett R.M., Eifler M.R., Zuleta A.M., Bailey S.C. (2022). Trajectories of perceived susceptibility to COVID-19 over a year: The COVID-19 & chronic conditions (C3) cohort study. Medicine.

[57] Al-Hanawi M.K., Ahmad K., Haque R., Keramat S.A. (2021). Willingness to receive COVID-19 vaccination among adults with chronic diseases in the Kingdom of Saudi Arabia. J. Infect. Public Health.

[58] Ellis S., Rosenblum K., Miller A., Peterson K.E., Lumeng J.C. (2014). Meaning of the terms “overweight” and “obese” among low-income women. J. Nutr. Educ. Behav..

